# Cardiac Magnetic Resonance Speckle Tracking Analysis of Right Ventricle Function in Myocarditis with Preserved Right Ventricular Ejection Fraction

**DOI:** 10.3390/medicina60101569

**Published:** 2024-09-25

**Authors:** Özge Özden, Serkan Ünlü, Ahmet Anıl Şahin, Ahmet Barutçu, Elif Ayduk Gövdeli, Sara Abou Sherif, Konstantinos Papadopoulos, Gülsüm Bingöl, Ismail Doğu Kılıç, Emre Özmen, Özden Seçkin Göbüt, Federico Landra, Matteo Cameli, Ömer Göktekin

**Affiliations:** 1 Department of Cardiology, Memorial Bahcelievler Hospital, Istanbul 34100, Turkey; ozgeozdenctf@hotmail.com (Ö.Ö.); bulut_gulsum@hotmail.com (G.B.); ogoktekin@gmail.com (Ö.G.); 2 Department of Cardiology, Gazi University Faculty of Medicine, Ankara 06500, Turkey; unlu.serkan@gmail.com; 3 Department of Cardiology, Istinye University, Liv Bahcesehir Hospital, Istanbul 34517, Turkey; aanilsahin@hotmail.com; 4 Department of Cardiology, Onsekizmart University Medical Faculty, Canakkale 17020, Turkey; ahmetbrtc@gmail.com; 5 Department of Cardiology, Royal Brompton & Harefield NHS Foundation Trust, London SW3 6PY, UK; elifayduk@gmail.com; 6 Cardiovascular Research Division, Kings College London, London SE5 9RS, UK; sara_sherif@hotmail.com; 7 Echocardiography Laboratory, European Interbalkan Medical Center, 555 35 Thessaloniki, Greece; papadocardio@gmail.com; 8 Department of Cardilogy, Pamukkale University Hospital, Denizli 20160, Turkey; idogukilic@yahoo.com; 9 Cardiology Department, Siirt Traing and Teaching Hospital, Siirt 56000, Turkey; dremreozmen@yahoo.com; 10 Deparment of Medical Biotechnologies, Division of Cardiology, University of Siena, 53100 Siena, Italy; f.landra@student.unisi.it (F.L.); matteo.cameli@yahoo.com (M.C.)

**Keywords:** acute myocarditis, right ventricular function, speckle tracking, cardiac magnetic resonance imaging

## Abstract

*Background and Objectives*: Diagnosis of myocarditis remains a challenge in clinical practice; however, magnetic resonance imaging (CMRI) can ease the diagnostic approach by providing various parameters. The prevalence of right ventricular involvement in acute myocarditis is suggested to be more frequent than previously hypothesized. In this study, we sought to investigate subclinical RV involvement in patients with acute myocarditis and preserved RV ejection fraction (EF), using CMRI RV speckle-tracking imaging. *Materials and Methods*: CMRI of 27 patients with acute myocarditis (nine females, age 35.1 ± 12.2 y) was retrospectively analyzed. A control group consisting of CMRI images of 27 healthy individuals was included. *Results*: No significant differences were found regarding left ventricle (LV) and atrium dimensions. LV ejection fraction was significantly different between groups (56.6 ± 10.6 vs. 62.1 ± 2.6, *p* < 0.05). No significant differences were present between parameters used for conventional assessment of RV. However, RV strain absolute values were significantly lower in the acute myocarditis group in comparison with that of the control group (18.4 ± 5.4 vs. 21.8 ± 2.8, *p* = 0.018). *Conclusions*: Subclinical RV dysfunction detected by CMR-derived strain may be present in patients with acute myocarditis even with preserved RVEF.

## 1. Introduction

Myocarditis, also known as inflammatory myocardial disease, poses a significant threat to cardiac health, contributing to both mortality and morbidity [1]. Its clinical presentation can vary widely, ranging from being asymptomatic to manifesting as a spectrum of conditions, including symptoms resembling a heart attack, heart failure, arrhythmias, and even sudden cardiac death [2,3]. Diagnosing myocarditis has historically been a clinical challenge. However, the advent of cardiac magnetic resonance imaging (CMRI) has greatly aided in this regard. CMRI provides a single comprehensive scan that reveals various myocarditis-related features such as contractile dysfunction, inflammatory hyperemia/edema, necrosis/scar, and the presence of pericardial effusion [4,5]. Innovative techniques like speckle tracking analysis offer potential supplementary information for identifying subclinical involvement. Until recently, right ventricular (RV) involvement in myocarditis was primarily documented in isolated case reports [6]. This could be attributed to the inherent complexities in assessing the RV’s anatomy and function using echocardiography. However, earlier endomyocardial biopsy studies countered this notion, showing that RV involvement in myocarditis was not uncommon [7]. Recent CMRI studies corroborate these findings, revealing that RV involvement in acute myocarditis is more frequent than previously believed [6].

In this study, our objective was to explore subclinical RV involvement in patients with acute myocarditis who maintain a preserved RV ejection fraction (EF). We employed CMRI RV speckle-tracking imaging for this investigation.

## 2. Methods

In this retrospective study, conducted between February 2020 and July 2022 at Memorial Bahçelievler Hospital and Çanakkale 18 Mart University Medical Faculty, we meticulously analyzed CMRI data from 28 consecutive patients previously diagnosed with acute myocarditis.

### 2.1. Patient Selection and Exclusions

We focused on a cohort of 28 patients initially suspected of having acute myocarditis based on clinical criteria. All patients with a preliminary or confirmed diagnosis of acute myocarditis underwent cardiac MRI imaging within one month at the latest. Exclusion criteria included patients under the age of 18 and those with pre-existing known cardiac diseases. We also excluded patients exhibiting impaired left ventricular (LV) or RV EF and RV edema, as detected through T2-STIR and native T2 mapping sequences, to ensure a homogeneous study group. Additionally, we included a control group consisting of 27 individuals who matched the study group in terms of age and sex and had normal CMRI results for comparative analysis. Ethical approval for our study protocol was granted by the local ethics committee, ensuring adherence to ethical guidelines in research involving human subjects.

### 2.2. Diagnostic Criteria for Myocarditis

Following the guidelines outlined in the latest position paper of the European Society of Cardiology [8], we considered patients with chest pain, new electrocardiogram (ECG) changes, elevated troponin levels, and wall motion abnormalities at echocardiography as suspected cases of myocarditis. A definitive diagnosis of myocarditis was established when the Lake Louise criteria [9], a set of CMRI-based diagnostic criteria, were met.

**Figure 1:**
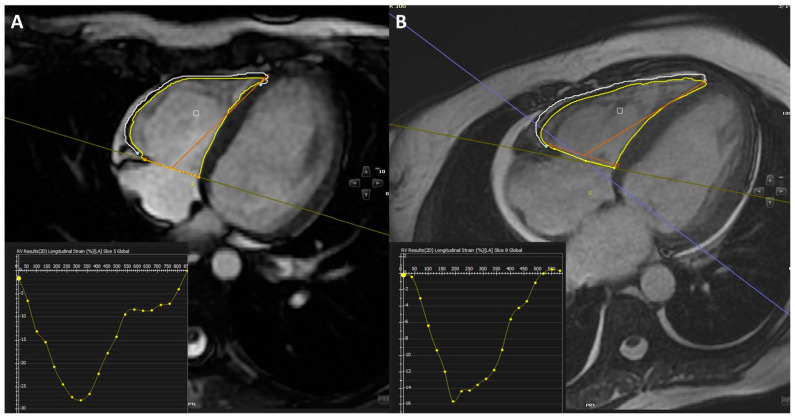
Example of feature tracking derived right ventricular longitudinal strain: (**A**) 4-chamber view of a patient with normal right ventricular longitudinal strain, (**B**) 4-chamber view of a patient with impaired right ventricular longitudinal strain.

All CMR examinations were performed using a 1.5 T scanner (Magnetom Avanto, Siemens Healthcare, Erlangen, Germany) at Memorial Bahçelievler Hospital and Çanakkale 18 Mart University Medical Faculty. CMRI protocols and slice planning followed standardized procedures, including steady-state free-precession (SSFP) for cine imaging at rest, triple-inversion T2-weighted spin-echo imaging for assessing myocardial edema, and contrast-enhanced spin-echo imaging for evaluating myocardial hyperemia, among others. Quantitative and qualitative offline assessments were carried out using dedicated software (CVI 42, version 5.1, Circle Cardiovascular Imaging, Calgary, AB, Canada). The presence of RV edema and fibrosis in T2-STIR and parametric T2 mapping images, as well as late gadolinium enhancement (LGE) images, was used to assess RV myocarditis. These images were cross-referenced with cine-SSFP images for a comprehensive evaluation. Global longitudinal strain data for RV were obtained through feature tracking evaluation using the Strain Module of CVI42. An example of strain analysis is provided in Figure 1.

### 2.3. Statistics

Continuous variables are presented as mean ± standard deviation or median with interquartile range, as appropriate. Categorical data are presented as percentages or frequencies. The Kolmogorov–Smirnov test was used to check for the normality of distribution for continuous variables. Student *t*-test and Mann–Whitney U test were used to compare parametric and nonparametric continuous variables, respectively. Categorical variables were compared by chi-square (χ^2^) test. A two-tailed *p*-value of <0.05 was considered as statistically significant. All data were analyzed using SPSS v23.0 (IBM Corp, Armonk, NY, USA). The G∗Power version 3.1.9.4 program was used to calculate the sample size. The results of the study by Di Bella G et al. and Meindl C were used to calculate the minimum required sample size [10,11]. The sample size to detect an effect size of 0.60 with a two-sided type I error rate of 5% and 90% power was estimated to be 20 individuals.

## 3. Results

The baseline characteristics of the patient group are given in Table 1. The patient population consisted of young adults, and the control group individuals were chosen accordingly. The mean age of the patient and the control groups were 35.1 ± 12.2 and 34.2 ± 9.8, respectively (*p* = 0.780). The majority of the population was male, showing no difference between groups (19 (67.8%) vs. 14 (51.9), *p* = 0.277). Chest pain was the most frequent symptom at first admission (71.4%). Other symptoms causing hospital admission were palpitations and dyspnea, both with a rate of 14.3%. All patients presented in the 1st week from the onset of symptoms and with high troponin levels and underwent either invasive coronary angiography (82.1%) or coronary computerized tomography angiography (CCTA) (17.9%) before CMRI to exclude myocardial infarction. Moreover, 10.7% of the patients had myocarditis shortly after COVID-19 vaccination.

As shown in Table 2, there were no significant differences in LV and atrium basic measurements except LVEF. Mean LVEF was 56.6 ± 10.6 in the myocarditis group, while it was 62.1 ± 2.6 in the control group (*p* < 0.05). There were no significant differences between RV and atrium parameters either. However, RV strain absolute values were significantly lower in the acute myocarditis group in comparison with that of the control group (*p* = 0.018).

## 4. Discussion

In this study, we investigated patients with acute myocarditis and assessed their CMRI and CMRI feature tracking (CMRI-FT) findings. Our findings revealed that patients with myocarditis exhibited subclinical RV dysfunction, as diagnosed through CMRI-FT imaging, even though they had preserved RV EF. Additionally, CMRI and CMRI-FT analysis demonstrated that patients with myocarditis had significantly lower LVEF and, more notably, impaired RV strain. However, there were no significant differences in terms of RV EF, biventricular volumes, biatrial area, tricuspid annular plane systolic excursion (TAPSE), and mitral annular plane systolic excursion (MAPSE) when compared with the control group, which was already matched for sex and age and included no patients with comorbidities.

CMR is widely recognized as the gold standard method for biventricular volume and function analysis, and it stands as the preferred diagnostic tool for myocarditis due to its unique ability to detect inflammatory changes in the myocardium. CMR-FT imaging, a novel technique that leverages cine images, provides additional valuable information for identifying subclinical biventricular dysfunction [12]. Notably, a study by Fischer et al. [13] demonstrated that myocardial strain analysis using CMRI-FT, in addition to conventional CMRI parameters, offered independent and incremental prognostic value over clinical features, LV EF, and LGE in patients with myocarditis. In our study, we evaluated myocardial strain through CMRI-FT in conjunction with conventional CMRI parameters in our patient cohort. Myocarditis remains a significant contributor to cardiac mortality and morbidity. While CMRI has been instrumental in diagnosing myocarditis, challenges persist in comprehensively studying the inflammatory process’s impact on the atria and ventricles. Previous research has predominantly focused on left ventricular (LV) strain analysis in myocarditis patients. For instance, studies by Chen et al. [14] and Lee et al. [15] highlighted the association of LV strain indices with early LV dysfunction and major cardiovascular adverse events. Consistent with these findings, our study also revealed significantly lower LV EF in the myocarditis group compared to the control group (LVEF 56.6% vs. 62.1%, *p*-value 0.035, respectively).

The assessment of RV function poses unique challenges but holds immense clinical relevance due to its critical role in cardiac and pulmonary physiology. Impaired RV function is widely recognized as a predictor of poor clinical outcomes in various cardiovascular conditions [10,16,17]. However, limited data exist regarding RV function and its post-myocarditis outcomes. Echocardiography studies, such as one by Khanna et al. [17], have reported reduced RV fractional area change and RV-free wall global longitudinal strain in patients with acute myocarditis. It is demonstrated that decreased baseline RV function in myocarditis patients compared to follow-up indicates that myocarditis can cause persistent systolic ventricular dysfunction despite eventual recovery [18,19,20]. Wang et al. also noted that while RV size improved over time, RV strain indices remained decreased in a three-month period [21]. In our study, we observed no differences in RV functional parameters, including TAPSE, RV volumes, and RVEF, between the two groups; however, RV strain indices were significantly lower in patients with myocarditis compared to controls (RV strain −18.4% vs. −21.8%, *p*-value 0.018, respectively). These findings challenge our initial hypothesis that RV function remains unimpaired during myocarditis, even when RVEF is preserved. Several factors may contribute to RV involvement in myocarditis, with some cases suggesting isolated RV involvement [22,23]. Thus, the importance of detecting RV involvement, particularly in patients with preserved LV function, becomes paramount during the diagnostic process [11]. Additionally, inflammation affecting the LV and LV dysfunction may also coincide with RV impairment, regardless of the presence of RV inflammation [24].

### Limitations

This study was conducted with a retrospective design and a relatively modest sample size of 55 participants recruited from two distinct medical centers. It is essential to recognize that the limited number of patients might impact the generalizability of the findings and potentially constrain the ability to establish definitive conclusions. Furthermore, due to the study’s design, there was an absence of follow-up CMRI scans and clinical assessments for patients diagnosed with myocarditis. Consequently, the long-term prognostic implications associated with RV strain values and changes in cardiac chamber dimensions remained unexplored. Additionally, there will be definitely a need for a new similar study design with long term follow-up to check if there is a normalization of RV speckle tracking values in myocarditis with preserved EF patient group. This is a noteworthy limitation, as it could offer valuable insights into the trajectory of myocarditis. The primary focus of this study was on RV strain analysis utilizing CMRI -FT imaging. While this approach provides valuable information, a more comprehensive evaluation of cardiac involvement in myocarditis could have been achieved by including analyses of LV global and regional myocardial strain, as well as left atrial strain. Such assessments could have contributed to a better understanding of diastolic function and the broader spectrum of cardiac implications in myocarditis. An additional limitation pertains to the lack of comprehensive data from other diagnostic modalities, such as echocardiography, and laboratory findings, which includes the absence of results from endomyocardial biopsy. This limitation arises from the specific role of our center as a reference for CMRI, leading to referrals that primarily target CMRI evaluations. Acknowledging these constraints is essential for a nuanced interpretation of the study’s outcomes. Despite these constraints, our study provides valuable insights into the realm of myocarditis and underscores the imperative for further investigations to both address these limitations and broaden our insights into this complex medical condition.

## 5. Conclusions

Subclinical RV dysfunction detected by CMR-derived strain may be present in patients with acute myocarditis even with preserved RV EF. Whether this finding also brings a prognostic value in terms of adverse events at follow-up has to be investigated in further studies.

## Figures and Tables

**Table 1:** Baseline characteristics of the patient group.

Parameters	Myocarditis Group (n = 28)	Control Group (n = 27)	*p*
Age (y)	35.1 ± 12.2	34.2 ± 9.3	0.787
Gender (f) (n, %)	9 (32.2)	13 (48.1)	0.277
Conventional coronary angiography (n, %)	23 (82.1)	-	-
Coronary angiography with computed tomography (n, %)	5 (17.9)	-	-
Pericardial effusion (n, %)	18 (64.3)	-	-
İndication for CMR			
Chest pain (n, %)	20 (71.4)	2 (7.4)	<0.001
Dyspnea (n, %)	4 (14.3)	-	-
Palpitation (n, %)	4 (14.3)	16 (59.3)	<0.001
COVID-19 vaccination	3 (10.7)	-	-
Suspected cardiac pathologies (n, %)	-	4 (14.8)	-
Suspected cardiac involvement in arrhythmic disorders (n, %)	-	3 (11.1)	-
Family history of dilated cardiomyopathy (n, %)	-	2 (7.4)	-

COVID-19; Coronavirus disease 2019.

**Table 2:** Cardiac magnetic resonance imaging findings of the study groups.

Parameters	Myocarditis Group (n = 28)	Control Group (n = 27)	*p*
**Left ventricle**			
LV EF (%)	56.6 ± 10.6	62.1 ± 2.6	0.035
Septal thickness (mm)	8 ± 2.6	8.7 ± 2.1	0.299
LV posterior wall thickness (mm)	7.3 ± 1.2	7.1 ± 1.6	0.537
LV EDD (mm)	50.9 ± 7.6	47.9 ± 3.9	0.106
LV ESD (mm)	36.8 ± 8.1	33.1 ± 3.8	0.069
MAPSE (mm)	12.2 ± 4.8	14.2 ± 2.2	0.096
RA area (cm^2^)	20.2 ± 3.8	20.8 ± 3.6	0.570
LA area (cm^2^)	19.8 ± 5.7	18.5 ± 5.5	0.446
LV EDVi (mL/m^2^)	85.9 ± 27	75.2 ± 12.2	0.088
LV ESVi (mL/m^2^)	39.3 ± 25.7	29 ± 6.3	0.096
**Right ventricle**			
RV EF (%)	56 ± 4.3	57.7 ± 3.2	0.129
TAPSE (mm)	21.2 ± 5	20.8 ± 4.2	0.775
RV EDV (mL)	77.9 ± 15.4	75.3 ± 15.1	0.568
RV ESV (mL)	35 ± 8.1	31.3 ± 7.8	0.140
RV strain (%)	18.4 ± 5.4	21.8 ± 2.8	0.018

EDD; end-diastolic diameter, EDV; end-diastolic volume, EDVi; end-diastolic volume index, ESD; end-systolic diameter, ESV; end-systolic volume, ESVi; end-systolic volume index, LA; left atrium; LV; left ventricle, RA; right atrium, RV; right ventricle.

## Data Availability

The data underlying this article will be shared upon reasonable request to the corresponding author.
